# Analysis of changes in the expression levels of peripheral blood immunoregulatory T Lymphocytes in children with bronchial asthma accompanied by recurrent infection

**DOI:** 10.12669/pjms.38.6.5521

**Published:** 2022

**Authors:** Junda Mi, Yong Guo

**Affiliations:** 1 Junda Mi, Department of Internal Medicine, The Third Outpatient Department, General Hospital of Northern Theater Command, Shenyang 110032, Liaoning, China; 2 Yong Guo, Department of Internal Medicine, The Third Outpatient Department, General Hospital of Northern Theater Command, Shenyang 110032, Liaoning, China

**Keywords:** Bronchial asthma, T lymphocyte subsets, Immunomodulatory T lymphocytes, Peripheral blood

## Abstract

**Objectives:**

To investigate the changes in the expression levels of peripheral blood immunoregulatory T lymphocytes in children with bronchial asthma accompanied by recurrent infection.

**Methods:**

Eighty children with bronchial asthma admitted to the Third Outpatient Department of General Hospital of Northern Theater Command from January 2017 to January 2019 were retrospectively selected and divided into two groups according to whether they had recurrent infection: the asthma group (n=49) and the recurrent infection group (n=31). Another 40 children in the same age group who had a health checkup or a surgical hospital preoperative examination were selected as the control group. Peripheral venous blood of each group was taken to measure the expression levels of T lymphocyte subsets (CD_3_^+^, CD_4_^+^, CD_8_^+^, CD_4_^+^/CD_8_^+^), and regulatory T cells (Treg) and helper T lymphocytes (Th1, Th2, Th17) in CD4+ subsets were detected at the same time. Moreover, Th1/Th2 values were calculated, and the levels of interleukin-4 (IL-4), interleukin-17 (IL-17), and interleukin-21 (IL-21) were measured.

**Results:**

The expression levels of CD3^+^ lymphocyte subsets were similar among the recurrent infection group, the asthma group, and the control group (P>0.05), and there were statistically significant differences in CD4^+^, CD8^+^ and CD4^+^/CD8^+^ lymphocyte subsets among the three groups (P<0.05). Statistically significant differences were observed in Treg, Th1, Th2, Th17 and Th1/Th2 values among the recurrent infection group, asthma group and control group (CD_3_^+^, CD_4_^+^, CD_8_^+^, CD_4_^+^/CD_8_^+^<0.05), and the expression levels of IL-4, IL-17 and IL-21 in the three groups also showed statistically significant differences (P<0.05).

**Conclusion:**

Bronchial asthma with recurrent infection may have a close bearing on the expression level of peripheral blood immunoregulatory T lymphocytes, and indirect examination of peripheral blood T lymphocyte level in children can be used clinically to determine the incidence of the disease, which is of high clinical application value.

## INTRODUCTION

Bronchial asthma, as one of the common chronic respiratory diseases in children, is a chronic inflammatory disease of the airway brought about by the participation of a variety of cells and cell components. In the wake of industrial development and worsening environmental pollution in recent years, bronchial asthma has witnessed a significant increase in the incidence, which in turn has a certain impact on the healthy growth of children.^1^ Bronchial asthma is complicated in pathogenesis with unknown etiology. It may be linked to various mechanisms such as neuroendocrine and immunity, and is mainly characterized by immune activation and immune imbalance.^2^ The pathological and physiological characteristics of bronchial asthma in children are the infiltration of inflammatory cells such as mast cells, eosinophils, lymphocytes, neutrophils and macrophages in the bronchial mucosa. Moreover, the characteristic inflammatory response of asthma is caused by T lymphocyte and eosinophil infiltration, accompanied by airway epithelial cell injury and airway structural remodeling.^3^

It has been confirmed in relevant studies that airway inflammation is also related to a variety of effector T lymphocyte subsets, and the number and function of its cell subsets may have a close bearing on the occurrence and progression of asthma, allergic rhinitis, etc.^4^ Taken into account this, changes in the expression levels of peripheral blood immunoregulatory T lymphocytes in bronchial asthma with recurrent infection were analyzed.

## METHODS

Eighty children with bronchial asthma admitted to the Third Outpatient Department of General Hospital of Northern Theater Command from January 2017 to January 2019 were retrospectively selected and divided into two groups according to whether they had recurrent infection: the asthma group (n=49) and the recurrent infection group (n=31).

### Inclusion criteria:

• Children who met the relevant diagnostic criteria of the “Guidelines for the Diagnosis and Prevention of Bronchial Asthma in Children (2016 Edition)”^5^ and were all in the onset of disease;
• Children who had taken relevant therapeutic drugs, including theophylline, leukotriene antagonist, glucocorticoid, etc., within two weeks before enrollment;
• Children whose clinical data and blood samples are complete, and whose chief guardian has informed consent;

### Exclusion criteria:

• Children with heart and lung diseases, including congenital heart disease, pulmonary embolism, and respiratory failure;
• Children with autoimmune diseases, including pemphigus, juvenile rheumatoid arthritis, systemic lupus erythematosus, etc.;
• Children who develop wheezing for the first time;
• Children with malignant tumors.

Another 40 children in the same age group who had a health checkup or a surgical hospital preoperative examination were selected as the control group. None of them had any history of other allergies (or family history of allergies), cardiopulmonary diseases, immune diseases or infectious diseases. No statistically significant difference was found in the general data between the groups (*P*>0.05), which was comparable.

### Ethical Approval

The study was approved by the Institutional Ethics Committee of The Third Outpatient Department, General Hospital of Northern Theater Command on February 1, 2017 (No.[2017]015), and written informed consent was obtained from all participants.

Two mililiter and 1mL of fasting peripheral venous blood were collected from the enrolled children twice in the morning by vacuum anticoagulant tube. Again two mililiter peripheral blood samples were taken for processing within four hour. The steps were as follows: post-stimulation of cells → labeling tube → fixation/rupture of membranes and cell perforation → intracellular immunofluorescence labeling, and then Beckman flow cytometry (Beckman-Coulter, Inc., United States) was utilized to detect the expression levels of T lymphocyte subsets (CD_3_^+^, CD_4_^+^, CD_8_^+^, CD_4_^+^/CD_8_^+^) in peripheral blood. Meanwhile, regulatory T cells (Treg) and helper T lymphocytes (Th1, Th2, Th17) in CD4^+^ subsets were detected, and Th1/Th2 value was calculated.

### Inflammatory factors

One mililiter peripheral blood was taken for one hour at room temperature, and the supernatant was taken after centrifugation and stored at -80°C. Enzyme-linked immunosorbent assay (ELISA) was used to measure the levels of inflammatory factors in the same group, including interleukin-4 (IL-4), interleukin-17 (IL-17), and interleukin-21 (IL-21). The expression levels of CD_3_^+^, CD_4_^+^, CD_8_^+^, CD_4_^+^/CD_8_^+^, Treg, Th1, Th2, Th17, Th1/Th2, IL-4, IL-17 and IL-21 in peripheral venous blood of children in each group were observed.

### Statistical Analysis

All data in this study were entered into EXCEl spreadsheet by two people without communication, and processed by statistical software SPSS17.0. Measurement data were expressed as Mean±SD (*x̅*±s). One-way ANOVA was used for comparison between multiple groups. Enumeration data were expressed by the number of cases (%), and disordered classification data were tested by χ2 tests. All tests were two-sided tests, and *P*<0.05 was considered statistically significant.

## RESULTS

The expression levels of CD_3_^+^ lymphocyte subsets were similar among the recurrent infection group, the asthma group, and the control group (*P*>0.05). The levels of CD_4_^+^ and CD_4_^+^/CD_8_^+^ lymphocyte subsets in children with bronchial asthma were significantly higher than those in control group, and CD_8_^+^ lymphocyte subsets were significantly lower than those in control group. The levels of CD_4_^+^ and CD_4_^+^/CD_8_^+^ in the recurrent infection group were significantly higher than those in the asthma group, and CD_8_^+^ levels were significantly lower than those in the asthma group. The differences were statistically significant (*P*<0.05).Table-II.

**Table I:** Comparison of general data among all groups.

Group	Gender [%]		Age (years old)

	Male	Female	
Asthma group (n=49)	25 (51.02)	24 (48.98)	8.33±2.57
Recurrent infection group (n=31)	18 (58.06)	13 (41.94)	8.42±2.69
Control group (n=40)	22 (55.00)	18 (45.00)	8.52±3.04
Χ^2^/F	0.396		0.052
P	0.820		0.949

**Table II:** Comparison of the expression levels of T lymphocyte subsets among all groups (*x̅*±s).

Group	CD_3_^+^ (%)	CD_4_^+^ (%)	CD_8_^+^ (%)	CD_4_^+^/CD_8_^+^
Asthma group (n=49)	64.84±12.33^a,c^	49.37±8.83^a,c^	32.14±4.35^a,c^	1.54±0.33^a,c^
Recurrent infection group (n=31)	63.15±15.57^b,c^	52.33±9.05^b,c^	30.68±5.22^b,c^	1.68±0.46^b,c^
Control group (n=40)	65.33±9.97^a,b^	43.32±7.54^a,b^	35.76±5.15^a,b^	1.25±0.34^a,b^
F	0.283	10.756	10.760	12.848
^a^ P	0.754	0.000	0.000	0.001
^b^ P	0.863	0.000	0.000	0.000
^c^ P	0.794	0.008	0.029	0.041

The levels of Th2 and Th17 in children with bronchial asthma were significantly higher than those in the control group, while Treg and Th1 levels and Th1/Th2 values were significantly lower than those in the control group. The levels of Th2 and Th17 in the recurrent infection group were significantly higher than those in the asthma group, while the levels of Treg and Th1 and Th1/Th2 were significantly lower than those in the asthma group. The differences were statistically significant (*P*<0.05). Table-III.

**Table III:** Comparison of the expression levels of regulatory T cells and helper T lymphocytes among all groups (*x̅*±s).

Group	Treg (%)	Th1 (%)	Th2 (%)	Th17 (%)	Th1/Th2
Asthma group (n=49)	5.89±1.76^a,c^	5.34±2.68^a,c^	4.05±1.97^a,c^	1.63±0.98^a,c^	1.97±0.58^a,c^
Recurrent infection group (n=31)	4.39±1.88^b,c^	4.76±2.21^b,c^	4.43±2.15^b,c^	1.74±1.12^b,c^	1.18±0.42^b,c^
Control group (n=40)	7.05±2.24^a,b^	6.28±2.55^a,b^	2.54±0.96^a,b^	0.95±0.54^a,b^	2.45±0.97^a,b^
F	16.050	3.343	12.316	8.707	28.508
^a^ P	0.000	0.011	0.000	0.000	0.002
^b^ P	0.000	0.008	0.000	0.000	0.000
^c^ P	0.005	0.039	0.027	0.044	0.005

The levels of IL-4 and IL-17 in children with bronchial asthma were significantly higher than those in the control group, while IL-21 levels were significantly lower than those in the control group. The levels of IL-4 and IL-17 in the recurrent infection group were significantly higher than those in the asthma group, while the levels of IL-21 were significantly lower than those in the asthma group. The differences were statistically significant (*P*<0.05). Table-IV.

**Table IV:** Comparison of the expression levels of inflammatory factors among all groups (*x̅*±s, ng/L).

Group	IL-4	IL-17	IL-21
Asthma group (n=49)	150.47±13.37^a,c^	5.35±2.14^a,c^	11.76±3.89^a,c^
Recurrent infection group (n=31)	184.46±20.55^b,c^	6.04±2.28^b,c^	9.54±4.42^b,c^
Control group (n=40)	65.54±10.38^a,b^	2.38±1.76^a,b^	17.54±2.84^a,b^
F	25.304	33.990	45.642
^a^ P	0.000	0.000	0.000
^b^ P	0.000	0.000	0.000
^c^ P	0.011	0.025	0.031

## DISCUSSION

Bronchial asthma, as a heterogeneous disease, is mainly characterized by chronic airway inflammation and airway hyperreactivity. Extensive and variable reversible airflow restriction occurs during the onset, inducing symptoms such as recurrent wheezing, shortness of breath, and cough, which mainly occur and worsen at night and/or early morning.^6^ Bronchial asthma occurs in all age groups, but it occurs frequently in childhood. In particular, it does serious harm to the health of children under the age of 14, and is prone to recurrent infection, which not only affects the normal study and life of the children, but also causes certain mental pressure and economic burden to their families.^7^

T lymphocytes are among the foremost cell groups for human immune function. Mature T cells can be divided into CD3^+^, CD4^+,^ and CD8^+^ lymphatic subsets according to different surface markers, in which CD3^+^ stands for T lymphocytes, CD4^+^ represents T helper cells, and CD8^+^ means T suppressor cells and T killer cells. Under normal circumstances, various cell subsets are in a state of dynamic equilibrium, maintaining normal cellular and humoral immune functions. When stimulated by allergens, the levels of CD4^+^ and CD8^+^ in the peripheral blood are obviously abnormal, in which the rise of CD4^+^ is late-onset asthma, and the decline of CD8^+^ is rapid-onset asthma.^8^,^9^ In this study, peripheral blood T lymphocyte subsets of children with bronchial asthma accompanied by recurrent infection were investigated. It was found that the CD3^+^ level during the onset of the disease was comparable to that of asthma and healthy children, while CD4^+^ level and CD4+/CD8^+^ value increased significantly, and CD8^+^ level was in a state of decline, which was similar to the results of Miao Q et al.^10^, suggesting that the number and function of T lymphocyte subsets in children with bronchial asthma accompanied by recurrent infection are in a state of dysfunction, and the number of T helper cells is dominant, while the number of inhibitory T cells is significantly less.

CD4^+^ lymphoid subsets are subdivided into Treg cells and Th cells according to different cell functions. To be specific, Treg cells can inhibit the proliferation and activation of Th cells, thereby reducing the synthesis of pro-inflammatory factors and improving the inflammation of the respiratory tract to a certain extent.^11^ Th cells play a regulatory role in asthma attacks, in which, if Th1 subsets are decreased, Th1 cell function is weakened, and if Th2 subsets are increased, Th2 cell function is hyperactive. In case of Th1/Th2 imbalance, a slight excess of inflammatory factors will occur, which will further promote the onset of airway inflammation.^12^ Th17 has a certain pro-inflammatory effect as a new Th cell subset, which can induce a variety of cells to produce inflammatory factors.^13^ As shown in the results of this study, the peripheral blood CD4^+^ lymphatic subsets of children with bronchial asthma accompanied by recurrent infection showed low expression of Treg and Th1, high expression of Th2 and Th17, and a low Th1/Th2 ratio, which was similar to the results of Gao Y et al.^14^ Treg cells and Th cells were abnormally expressed in children with bronchial asthma accompanied by recurrent infection. This is similar to the results of Krishnamoorthy et al.,^15^ which may be due to the gradual development of immune tolerance during repeated infection that stimulates the expression of Treg cells.

As a chronic inflammatory airway disease, bronchial asthma is closely related to the type 2 cytokine interleukin, which promotes airway eosinophilia, excess mucus secretion, bronchial hyperresponsiveness and immunoglobulin E synthesis.^16^,^17^ In our study, the levels of IL-4 and IL-17 in children with bronchial asthma were significantly higher than those in the control group, the above indexes were significantly higher in recurrent infection group than in asthma group, which were similar to the results of previous studies.^18^,^19^ The levels of IL-21 in the recurrent infection group were significantly lower than those in the asthma group, which was inconsistent with the results of al-Ayed et al.^20^ The reason may be related to the small sample size of this study, and this study only grouped children with asthma according to whether they had recurrent infection or not, without considering some other possible influencing factors, such as obesity, which may produce bias.

### Limitations of this study

The number of subjects included in this study was limited, so the conclusions drawn may not be very convincing. In addition, this study only grouped children with asthma according to whether they had recurrent infection or not, without considering some other possible influencing factors, such as obesity, which may produce bias.

## CONCLUSION

Bronchial asthma accompanied by recurrent infection may have a bearing on abnormal expression of peripheral blood immunoregulatory T lymphocytes, in which CD4^+^, Th2, Th17, IL-4 and IL-17 significantly increased, while CD8^+^, Treg, Th1 and IL-21 significantly decreased, CD4^+^/CD8^+^ ratio increased, and Th1/Th2 ratio decreased. All the above indicators are closely related to the development of asthma, thus providing fresh ideas for clinical diagnosis and treatment of bronchial asthma.

### Authors’ Contributions:

**JM:** Designed this study, prepared this manuscript, are responsible and accountable for the accuracy and integrity of the work.

**YG:** Collected and analyzed clinical data, significantly revised this manuscript.
